# The correlation of CT-derived muscle density, skeletal muscle index, and visceral adipose tissue with nutritional status in severely injured patients

**DOI:** 10.1007/s00068-024-02624-6

**Published:** 2024-08-21

**Authors:** Elaine P. X. van Ee, Esmee A. H. Verheul, Suzan Dijkink, Pieta Krijnen, Wouter Veldhuis, Shirin S. Feshtali, Laura Avery, Claudia J. Lucassen, Sven D. Mieog, John O. Hwabejire, Inger B. Schipper

**Affiliations:** 1 Department of Trauma Surgery, Leiden University Medical Center, Post zone K6-R|, P.O. Box 9600, Leiden, 2300 RC The Netherlands; 2 Trauma, Emergency Surgery, and Surgical Critical Care, Massachusetts General Hospital, Harvard Medical School, Boston, MA USA; 3 Department of Surgery, Haaglanden Medical Center, The Hague, The Netherlands; 4 Acute Care Network West Netherlands, Leiden, the Netherlands; 5 Department of Radiology, University Medical Center Utrecht, Utrecht, The Netherlands; 6 Department of Radiology, Leiden University Medical Center, Leiden, The Netherlands; 7 Department of Radiology, Massachusetts General Hospital, Harvard Medical School, Boston, MA USA; 8 Department of Dietetics, Leiden University Medical Center, Leiden, The Netherlands; 9 Department of Surgery, Leiden University Medical Center, Leiden, The Netherlands

**Keywords:** Trauma, Critical care, Multicenter, Nutrition, Nutritional status

## Abstract

**Background:**

This study explored if computerized tomography-derived body composition parameters (CT-BCPs) are related to malnutrition in severely injured patients admitted to the Intensive Care Unit (ICU).

**Methods:**

This prospective cohort study included severely injured (Injury Severity Score ≥ 16) patients, admitted to the ICU of three level-1 trauma centers between 2018 and 2022. Abdominal CT scans were retrospectively analyzed to assess the CT-BCPs: muscle density (MD), skeletal muscle index (SMI), and visceral adipose tissue (VAT). The Subjective Global Assessment was used to diagnose malnutrition at ICU admission and on day 5 of admission, and the modified Nutrition Risk in Critically ill at admission was used to assess the nutritional risk.

**Results:**

Seven (11%) of the 65 analyzed patients had malnutrition at ICU admission, increasing to 23 patients (35%) on day 5. Thirteen (20%) patients had high nutritional risk. CT-BCPs were not related to malnutrition at ICU admission and on day 5. Patients with high nutritional risk at admission had lower MD (median (IQR) 32.1 HU (25.8–43.3) vs. 46.9 HU (37.7–53.3); *p* < 0.01) and higher VAT (median 166.5 cm^2^ (80.6–342.6) vs. 92.0 cm^2^ (40.6–148.2); *p* = 0.01) than patients with low nutritional risk.

**Conclusion:**

CT-BCPs do not seem related to malnutrition, but low MD and high VAT may be associated with high nutritional risk. These findings may prove beneficial for clinical practice, as they suggest that CT-derived parameters may provide valuable information on nutritional risk in severely injured patients, in addition to conventional nutritional assessment and screening tools.

**Level of Evidence:**

Level III, Prognostic/Epidemiological.

## Introduction

Malnutrition is associated with an increased risk of complications, mortality, prolonged hospital length of stay, and reduced quality of life in severely injured patients [1]. The prevalence of malnutrition in this patient group ranges from 7 to 76%, depending upon the setting, population, and nutritional assessment tool used [1]. However, objective in-hospital measurement of the nutritional status of severely injured patients remains a challenge, since obtaining their dietary history is often hampered by a decreased level of consciousness or mechanical ventilation. Evaluation of muscle wasting can be misleading due to swelling and oedema, and serum concentrations of visceral proteins are affected by the acute-phase response after inflammation or trauma [2–4]. 

Computerized tomography derived body composition parameters (CT-BCPs) may be helpful for the assessment of nutritional status of severely injured patients [5]. CT scans are routinely obtained of severely injured patients at admission, and can potentially serve as a new way of assessing body composition [6]. The CT-BCPs include muscle density (MD), skeletal muscle index (SMI), and visceral adipose tissue (VAT), and are assessed through abdominal CT scan analysis. MD reflects the density of muscles to indicate intramuscular fat accumulation and therefore muscle quality: a higher MD correlates with better muscle quality [7, 8]. The SMI is the skeletal muscle mass normalized for the patient’s height [9]. In oncology patients, a low SMI is found to be associated with malnutrition [9]. VAT defines the visceral adipose tissue surrounding the intra-abdominal organs [7]. A lower VAT is related to malnutrition in patients with gastric cancer [10]. These CT-BCPs have been proven indicative of nutritional status in several patient populations, such as those with Crohn’s disease, and malignancies, [9–12] but have not yet been studied in relation to nutritional status of severely injured patients.

This study aimed to examine the value of CT-BCPs (MD, SMI, and VAT) as assessment tools for malnutrition and nutritional risk in severely injured patients admitted to the ICU.

## Methods

### Study design and population

This exploratory cohort study is based on both prospectively and retrospectively collected data at one level-1 trauma center in the United States, and two Level-1 trauma centers in the Netherlands. The study is part of the prospective Malnutrition in Polytrauma Patients (MaPP) study, which was initiated in July 2018 [13]. In the MaPP study, patients were included if they: (i) were aged ≥ 18 years; (ii) had an Injury Severity Score (ISS) ≥ 16, due to blunt trauma; and (iii) were admitted to the Intensive Care Unit (ICU) at a participating hospital within six hours after trauma and for a period longer than 48 h between July 2018 and April 2022. Patients were excluded if they: (i) were transferred from another hospital to a participating center; or (ii) had burn wounds or penetrating injuries. Informed consent was obtained from the patients or their legal representative on the day of ICU admission or as soon as possible thereafter. In total, 100 patients were included in the MaPP study.

For the present exploratory substudy, no sample size was calculated. The substudy included all 65 MaPP study patients with an abdominal CT scan made for trauma assessment at admission to the emergency department of three of the five MaPP-centers. The abdominal CT scan data was analyzed retrospectively. Since 91 of 100 MaPP patients were admitted to three of the five centers, it was decided to restrict this substudy to these three centers for practical reasons. Both the MaPP study and the present CT-BCPs substudy were approved by the local Institutional Review Boards of the participating hospitals.

### Study parameters and definitions

Baseline data, such as medical history, Injury Severity Score (ISS), height and weight, and clinical data during the hospital stay, were gathered prospectively in Castor Electronic Data Capture (EDC) [14]. 

### CT derived body composition parameters

Abdominal CT scans with intravenous contrast, 120 kV, and coupe thickness of 5 mm on the day of ICU admission were retrospectively analysed using Quantib-U [15, 16]. This software program automatically marks and quantifies different tissues, such as muscle and visceral adipose fat [15, 16]. CT-BCPs included MD (in Hounsfield Units (HU)), SMI (in cm^2^/m^2^), and VAT (in cm^2^). MD and VAT were determined by analysis of single-slice axial abdominal CT scans at the top of the third lumbar vertebra (L3 level) [15, 17]. 

**Fig. 1:**
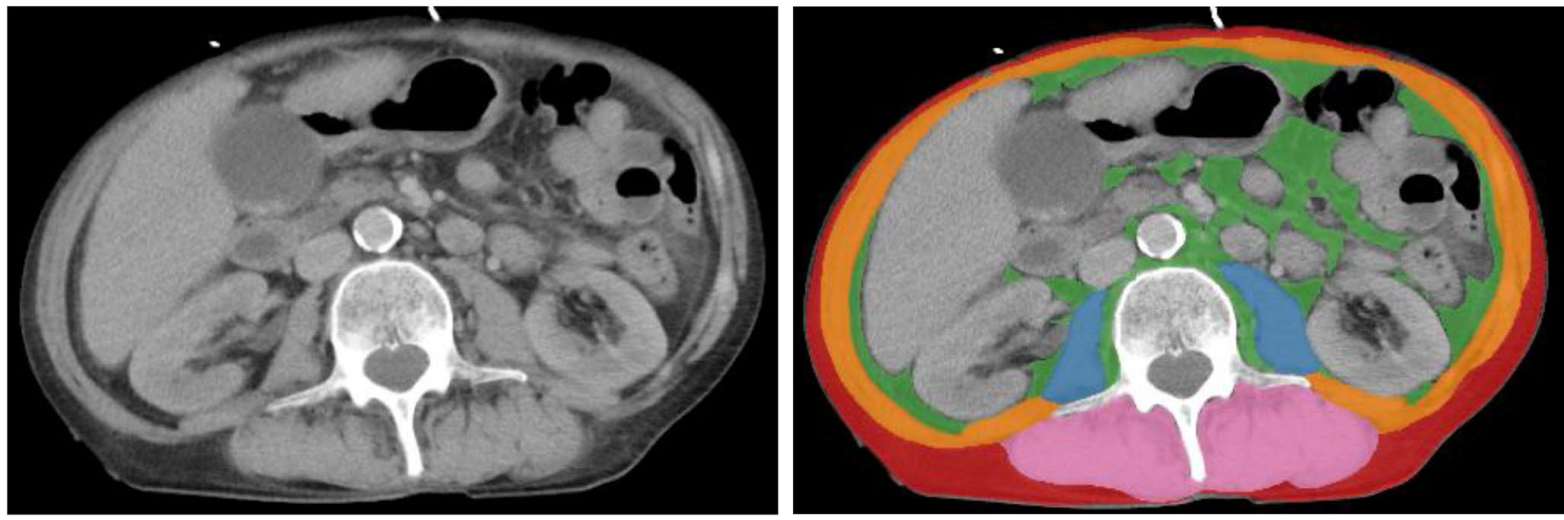
Example of an area analysis of CT-BCPs on the L3 level of a CT scan. Measurement of visceral adipose tissue area: 59.1 cm^2^ (green), subcutaneous adipose tissue area: 52.9 cm^2^ (red), psoas muscle area: 17.7 cm^2^ (blue), abdominal muscle area: 61.6 cm^2^ (orange), long spine muscle area: 51.5 cm^2^ (pink)

The mean radiation attenuation of the complete muscle area at the top L3 level was calculated from contrast-enhanced CT scans to assess the skeletal muscle density [18]. MD was calculated as the mean density of the skeletal muscle area (SMA) of the following muscle groups; musculus rectus abdominis, musculus transverses, musculus obliquus internus, musculus obliquus externus, musculus psoas major and minor, musculus erector spinae and musculus quadratus lumborum, and was expressed in HU [17]. To adjust for height, SMI was calculated by dividing the SMA (in cm^2^) by the square of the height of the patient (in m^2^), and was therefore expressed in cm^2^/m^2^ [19]. VAT was determined by staining the visceral adipose tissue at the L3 level with the use of Quantib-U (in cm^2^). Corresponding HU were − 29 to + 150 for SMA and − 50 to -150 for VAT [7, 20]. In Fig. 1, an example of area measurement at the L3 level is presented.

### Malnutrition

Malnutrition was assessed with the Subjective Global Assessment (SGA) score. The SGA score can be used to diagnose malnutrition, and was developed in surgical patients [21, 22]. It includes weight change (over the past 2 weeks and past 6 months), dietary intake change, gastrointestinal symptoms (less appetite, nausea, vomiting, diarrhoea), and functional capacity (difficulty with normal activities, dysfunction, bedridden). In addition, the SGA score includes a physical examination of muscle wasting (e.g. clavicle, knee, shoulder, and quadriceps), and subcutaneous fat loss (eyes, biceps, triceps). The SGA score ranges from 1 to 7, with 1–5 indicating malnutrition, and score 6–7 indicating no malnutrition [22]. SGA was scored by a trained nurse or member of the research group by using information provided by the patient him/herself, or, if this was not possible, by using information provided by family or the partner.

For this study, the nutritional status at ICU admission and day 5 of ICU admission were determined.

### Nutritional risk

The modified Nutrition Risk in the Critically Ill (mNUTRIC) scale was assessed by trained personnel within 24 h after ICU admission [21]. It identifies critically ill patients who are most likely to benefit from aggressive nutritional treatment and is the first nutritional risk assessment tool developed and validated specifically for critically ill patients [23, 24]. The mNUTRIC score is based on age, the Acute Physiology And Chronic Health Evaluation (APACHE II) score, the Sequential Organ Failure Assessment (SOFA) score, the number of comorbidities, and the number of days in the hospital before ICU admission [23, 25]. The APACHE II score is used as a general measure of the severity of the disease, and the SOFA score provides information about the prognosis of critically ill patients [26, 27]. The mNUTRIC score ranges from 1 to 9 is, and a score ≥ 5 is regarded as high nutritional risk [24]. 

### Statistical analysis

Baseline characteristics of patients with and without malnutrition on day 5 and of patients with high and low nutritional risk were compared using the Chi-square test or Fisher’s exact test for categorical variables, the independent samples T-test for normally distributed continuous variables, and the Mann-Whitney U test for skewed continuous variables. The Mann-Whitney U test was also used for all CT-BCP analyses.

First, to assess the value of CT-BCPs in diagnosing malnutrition, the CT-BCP levels in patients with and without SGA-diagnosed malnutrition at ICU admission were compared. Second, to evaluate whether CT-BCPs are predictive for developing malnutrition, the CT-BCPs were compared in the groups with and without malnutrition at day 5 of ICU admission. Third, the CT-BCP levels in patients with high and low nutritional risk (according to mNUTRIC score) were compared.

P-values of < 0.05 were considered statistically significant. All analyses were performed using IBM SPSS Statistics for Windows, version 25 (IBM Corp., Armonk, N.Y., USA).

## Results

### Study population

Seven of the 65 analyzed patients (11%) were malnourished at ICU admission, which increased to 23 patients (35%) on day 5 of admission. Characteristics of the 65 patients grouped according to their nutritional status on day 5 are presented in Table 1. The malnourished and well-nourished patients were comparable regarding baseline characteristics. The mean age was 48.9 (± 19.0) years, and 66% were male. Eight (12%) patients died during hospital admission, of which five died during ICU admission, and three after being admitted to the ward.

**Table 1:** Patient characteristics grouped according to their nutritional status on day 5 of admission

	Total (*n* = 65)	Well-nourished (SGA ≤ 5) (*n* = 42)	Malnourished (SGA > 5) (*n* = 23)	*P* value
Age in years, mean ± SD	48.9 ± 19.0	46.2 ± 17.0	54.0 ± 21.8	0.12
Male sex	43 (66)	29 (69)	14 (61)	0.59
APACHE II score, median (IQR)	17.0 (11.0–20.0)	16.0 (10.0-19.3)	18.0 (12.0–21.0)	0.19
SOFA score at admission, median (IQR)	7.0 (4.0–9.0)	7.0 (4.0–10.0)	6.0 (4.0–8.0)	0.32
BMI category * Healthy weight (18.5–25.0)* * Overweight (25.0–30.0)* * Obese (≥ 30.0)*	29 (45) 22 (34) 14 (22)	17 (40) 16 (38) 9 (21)	12 (52) 6 (26) 5 (22)	0.58
Severe injury (AIS ≥ 4) * Head* * Chest* * Abdomen* * Extremity*	27 (42) 20 (31) 6 (9) 12 (19)	15 (36) 16 (38) 3 (7) 10 (24)	12 (52) 4 (17) 3 (13) 2 (9)	0.29 0.10 0.66 0.19
ISS ≥ 25	46 (71)	32 (76)	14 (61)	0.26
GCS score ≤ 8	30 (46)	20 (48)	10 (43)	0.80
> 1 Comorbidity *	26 (40)	14 (33)	12 (52)	0.19

n(%) unless stated otherwise

AIS, Abbreviated Injury Scale; APACHE II, Acute Physiology and Chronic Health Evaluation; BMI, Body Mass Index; GCS, Glasgow Coma Scale; ICU, Intensive Care Unit; IQR, Interquartile range; ISS, Injury Severity Score; kg, kilograms; LOS, Length of Stay; n, number; SD, standard deviation; SGA, Subjective Global Assessment; SOFA, Sequential Organ Failure Assessment; y, years;

* According to mNUTRIC comorbidity list

Thirteen (20%) patients were considered to have high nutritional risk (mNUTRIC ≥ 5) at admission. The patients with high nutritional risk were significantly older (66.2 ± 10.5 vs. 44.6 ± 18.3 years; *p* < 0.001), suffered more often from a Glasgow Coma Scale (GCS) ≤ 8 (77% vs. 39%; *p* = 0.03), and had more often more than one comorbidity (92% vs. 27%; *p* < 0.001).

### Body composition parameters

The CT-BCP levels did not differ between the malnourished and the well-nourished patients at ICU admission (Table 2a), nor did CT-BCP levels differ between the malnourished and the well-nourished patients at day 5 of ICU admission (Table 2b).

**Table 2:** Median CT-BCPs levels (interquartile range) in patient groups according to their nutritional status (a) at admission and (b) on day 5

Nutritional status (SGA score)			
	Well-nourished	Malnourished	P value
(a) At admission	*n* = 58	*n* = 7	
MD	43.6 (35.7–51.9)	45.4 (29.6–53.0)	0.82
SMI	50.1 (41.5–58.5)	40.3 (35.9–59.6)	0.43
VAT	101.5 (52.7–155.7)	179.7 (39.0–235.7)	0.37
(b) On day 5	*n* = 42	*n* = 23	
MD	44.3 (36.6–52.0)	41.9 (26.9–50.5)	0.27
SMI	50.1 (41.5–59.1)	48.1 (38.6–58.3)	0.38
VAT	99.5 (51.7–157.4)	105.0 (54.1–185.5)	0.61

CT-BCPs, Computerized Tomography derived Body Composition Parameters; MD, Muscle Density (Hounsfield Units); mNUTRIC, modified Nutrition Risk in Critically ill; SGA, Subjective Global Assessment; SMI, Skeletal Muscle Index (cm^2^/m^2^); VAT, Visceral Adipose Tissue (cm^2^)

In the patients with high nutritional risk, the median MD was significantly lower than in the patients with low nutritional risk (32.1 HU, interquartile range (IQR) 25.8–43.3 vs. 46.9 HU, IQR 37.7–53.3; *p* < 0.01), and the median VAT was significantly higher (166.5 cm^2^, IQR 80.6–342.6 vs. 92.0 cm^2^, IQR 40.6–148.2; *p* = 0.01) (Table 3).

**Table 3:** Median CT-BCPs levels (interquartile range) in patient groups according to nutritional risk at admission

Nutritional risk (mNUTRIC score)			
	Low risk (*n* = 52)	High risk (*n* = 13)	P value
MD	46.9 (37.7–53.3)	32.1 (25.8–43.3)	**< 0.01**
SMI	48.4 (40.3–58.2)	51.5 (40.1–60.1)	0.67
VAT	92.0 (40.6–148.2)	166.5 (80.6–342.6)	**0.01**

CT-BCPs, Computerized Tomography derived Body Composition Parameters; MD, Muscle Density (Hounsfield Units); mNUTRIC, modified Nutrition Risk in Critically ill; SGA, Subjective Global Assessment; SMI, Skeletal Muscle Index (cm^2^/m^2^); VAT, Visceral Adipose Tissue (cm^2^)

## Discussion

This study aimed to examine the value of CT scan derived body composition parameters (MD, SMI, and VAT) as assessment tools for malnutrition and nutritional risk in severely injured patients admitted to the ICU. No correlation was found between the CT-BCPs and malnutrition as measured by the SGA on admission and at day 5 after admission. Low MD and high VAT were found to be related to high nutritional risk for severely injured patients that were admitted to the ICU.

Several studies have evaluated the relation between CT-BCPs and malnutrition in cancer patient populations. Malnourished patients undergoing radical gastrectomy for gastric cancer had lower SMI, MD, and VAT than the well-nourished patients [10]. In patients suffering from oesophageal and gastric cancer, malnourished patients had a lower SMI than well-nourished patients [9]. However, this relation was less evident in patients admitted to the ICU. In critically ill patients that require mechanical ventilation, MD and SMI seemed to be slightly lower in SGA-diagnosed malnourished patients, however not statistically significant [28]. This is comparable to our results, as we found that SMI tended to be lower in the malnourished patients compared to the well-nourished patients at ICU admission (40.3 (IQR 35.9–59.6) vs. 50.1 (41.5–58.5) cm^2^/m^2^; Table 2a), however not statistically significant (*p* = 0.43). In addition, the median VAT seemed higher in the malnourished patients at ICU admission compared to the well-nourished patients, but also not statistically significant (179.7 (39.0–235.7) vs. 101.5 (52.7–155.7) cm^2^, *p* = 0.37; Table 2a). A possible explanation for not finding a relation between SGA-diagnosed malnutrition and CT-BCPs could be the increasing prevalence of obesity [28]. When the SGA was developed in 1982, nutritional assessment primarily entailed detection of obvious signs of muscle and fat wasting [22, 29]. However, when a person is overweight or obese, malnutrition may not immediately be recognized by the conventional nutritional assessment tools [28]. 

Previous studies have shown that the risk of malnutrition was related to high VAT values and low SMI values in patients undergoing surgery for colorectal cancer [11]. Our study did not find a relation between CT-BCPs and malnutrition at day 5 of admission (Table 2b). On the other hand, we did find that the severely injured patients with high nutritional risk had significantly lower MD and higher VAT values (Table 3). High levels of MD and SMI are desirable, while high levels of VAT are associated with impaired survival in critically ill patients [7, 30]. In addition, two large systematic reviews pointed out that sarcopenia, defined by the presence of both low muscle mass and low muscle function, and visceral adiposity were related to mortality [31, 32]. Since the mNUTRIC is also related to mortality, this could indirectly explain why our study found low MD and high VAT in the patients with high nutritional risk [33]. Furthermore, in contrast to the SGA, the mNUTRIC does not require physical examination and is thus not influenced by the increasing prevalence of obesity [23]. 

To conclude, body composition analysis can provide objective information about muscle and adiposity status, and that can enhance nutritional assessment in addition to the conventional nutritional assessment and screening tools. CT scans are routinely conducted on severely injured patients, which allows for easy integration of determination of CT-BCPs into clinical practice [6]. Efficient analysis of these parameters on body composition can be performed using artificial intelligence segmentation algorithms, such as the Quantib-U deep learning algorithm [15, 16, 34]. Calculating MD and VAT in severely injured patients can be a routine assessment that does not require additional diagnostic actions by healthcare professionals.

### Limitations

Several limitations of this study should be taken into consideration when interpreting its results. There is no gold standard or solid measure for the determination of the nutritional status of a patient. We used the SGA score, as it is validated for ICU patients and proven to be the most predictive for in-hospital outcomes. The difference between an SGA score of 6 (well-nourished) or 5 (malnourished) can, however, be minimal. To increase the reliability for this study, the SGA scores were verified by a second investigator after the completion of data collection. Furthermore, this study covered a prospective cohort with retrospectively analyzed CT scans. Several MaPP-study patients could not be included, due to missing or incorrectly produced CT scans. Standardizing trauma-CT scans will ensure their reliability and comparability across the entire study population and minimize the risk of errors or inconsistencies. Finally, the study’s sample size was small with a low number of patients that had an SGA of 5 or less. The low number may pose challenges in extrapolating the results. Due to the limited sample size, it was not feasible to incorporate other variables into the analysis and perform sub-analyses.

Future research could focus on collecting multiple CT scans at different time points in more extensive patient cohorts, enabling to study the changes in body composition within individuals more precisely. Including higher numbers of patients would allow for more in-depth analyses. It would probably also allow for the composition of prediction models on hospital parameters and functional outcomes related to CT-BCPs.

## Conclusion

This study did not find a correlation between CT derived body composition parameters and malnutrition itself. It did show an increased nutritional risk for severely injured patients with low CT measured muscle density or high visceral adipose tissue parameters. Given that CT scan are routinely conducted on severely injured patients, it allows for easy integration of these parameters in clinical practice as a routine assessment. In this way CT scans can provide valuable information on body composition in severely injured patients in addition to conventional nutritional assessment and screening tools, identifying those severely injured patients with increased risk of malnutrition at the moment of hospital admission.

## Data Availability

Data is provided within the manuscript.
